# Identification of Two Novel Anti-Fibrotic Benzopyran Compounds Produced by Engineered Strains Derived from *Streptomyces xiamenensis* M1-94P that Originated from Deep-Sea Sediments

**DOI:** 10.3390/md11104035

**Published:** 2013-10-22

**Authors:** Zhong-Yuan You, Ya-Hui Wang, Zhi-Gang Zhang, Min-Juan Xu, Shu-Jie Xie, Tie-Sheng Han, Lei Feng, Xue-Gong Li, Jun Xu

**Affiliations:** 1 State Key Laboratory of Microbial Metabolism and School of Life Science & Biotechnology, State Key Laboratory of Ocean Engineering, Shanghai Jiao Tong University, Shanghai 200240, China; E-Mails: you-zhong-yuan@163.com (Z.-Y.Y.); hts@sjtu.edu.cn (T.-S.H.); agong1983@163.com (X.-G.L.); 2 State Key Laboratory of Oncogenes and Related Genes, Shanghai Cancer Institute, Ren Ji Hospital, School of Medicine, Shanghai Jiao Tong University, Shanghai 200240, China; E-Mails: yinhe028@163.com (Y.-H.W.); zzhang@shsci.org (Z.-G.Z.); 3 Ministry of Education Key Laboratory of Systems Biomedicine, Shanghai Center for Systems Biomedicine, Shanghai Jiao Tong University, Shanghai 200240, China; E-Mail: minjuanxu@sjtu.edu.cn; 4 Key Laboratory of Marine Biogenetic Resources, the Third Institute of Oceanography SOA, Xiamen 361005, Fujian, China; E-Mail: xieshujie40@163.com; 5 Instrumental Analysis Center, Shanghai Jiao Tong University, Shanghai 200240, China; E-Mail: fiona.fenglei@sjtu.edu.cn

**Keywords:** *Streptomyces xiamenensis*, ribosome engineering, benzopyran, anti-fibrosis

## Abstract

The benzopyran compound obtained by cultivating a mangrove-derived strain, *Streptomyces xiamenensis* strain 318, shows multiple biological effects, including anti-fibrotic and anti-hypertrophic scar properties. To increase the diversity in the structures of the available benzopyrans, by means of biosynthesis, the strain was screened for spontaneous rifampicin resistance (Rif), and a mutated *rpsL* gene to confer streptomycin resistance (Str), was introduced into the *S. xiamenensis* strain M1-94P that originated from deep-sea sediments. Two new benzopyran derivatives, named xiamenmycin C (**1**) and D (**2**), were isolated from the crude extracts of a selected Str-Rif double mutant (M6) of M1-94P. The structures of **1** and **2** were identified by analyzing extensive spectroscopic data. Compounds **1** and **2** both inhibit the proliferation of human lung fibroblasts (WI26), and **1** exhibits better anti-fibrotic activity than xiamenmycin. Our study presents the novel bioactive compounds isolated from *S. xiamenensis* mutant strain M6 constructed by ribosome engineering, which could be a useful approach in the discovery of new anti-fibrotic compounds.

## 1. Introduction

During daily urban life, a causal relationship between the elevated urban air pollution and an increased severity of airway diseases, including lung fibrosis and lung cancers, has been supported by epidemiological and toxicological research [1,2]. Fibrosis, a result of chronic inflammatory reactions induced by a variety of stimuli, has recently garnered increasing attention [3]. Fibrotic diseases such as idiopathic pulmonary fibrosis, liver cirrhosis, systemic sclerosis, progressive kidney disease, and cardiovascular fibrosis are threatening the public health [3]. However, successful methods for treating fibrosis have been limited, and the lack of effective small-molecule medicines is one of the serious issues [4,5]. Therefore, the search for bioactive compounds from natural resources represents an emerging pharmacological and therapeutic area in the fight against excessive fibrotic diseases.

Benzopyran derivatives have been demonstrated to have considerable bioactivities, including anti-oxidative, anti-hypertensive, anti-microorganism, and anti-inflammatory properties [6,7,8,9]. In our previous studies, it was shown that xiamenmycin, a benzopyran compound with the structure of *N*-((3,4-dihydro-3*S*-hydroxy-2*S*-methyl-2-(4′*R*-methyl-3′*S*-pentenyl)-2*H*-1-benzopyran-6-yl)carbonyl)-threonine, was obtained by cultivating the mangrove-derived strain *Streptomyces xiamenensis* 318, and it was found to have multiple biological effects toward inhibiting fibrosis, *i.e.*, the inhibition of excessive lung fibrosis *in vitro* and the attenuation of hypertrophic scars by the suppression of local inflammation, and by the reduction of the effects of mechanical stress [10,11,12]. The possible mechanism could be the inhibition of the mechanical stress-induced pro-fibrotic effects by suppressing proliferation, activation, and contraction of fibroblast, and inactivating focal adhesion kinase (FAK), p38, and Rho guanosine triphosphatase signaling [10,11,12]. During excessive fibrogenesis, the existence of self-perpetuating loops for inflammation and extracellular matrix (ECM) accumulation, which results from the inflammatory response and the mechanical forces, is closely related to formation of fibrotic diseases [13]. Xiamenmycin, aimed at the loops, has inhibitory effects on both inflammation and mechanotransduction; therefore, it may be a potential candidate for the development of new anti-fibrotic drugs. Thus, in our ongoing study, it is worthwhile to continue the chemical and pharmaceutical investigations of new benzopyran compounds.

*Streptomyces* are known as versatile producers of novel secondary metabolites from various biosynthetic pathways. The same species of *Streptomyces* strains even if the strains originate from different ecological niches can be used to hunt for novel bioactive compounds. Not surprisingly, an affined strain of *S. xiamenensis* 318, namely the strain M1-94P isolated from deep-sea sediments, can produce small amounts of potentially novel benzopyrans. As the secondary metabolic pathways in the microorganisms isolated from the deep sea normally remained dormant or weakly expressed under laboratory conditions, rational strain development was critical prior to extensive chemical investigation. Ribosomal engineering is a simple but practical approach for strain breeding by targeting the microbial ribosomal proteins or the subunits of RNA polymerase and has been widely used for strain improvement [14,15,16,17]. This strategy was therefore adopted to either increase the production of the benzopyran compound or stimulate the biosynthesis of novel benzopyrans by activating dormant or weakly expressed secondary metabolite biosynthetic genes in the strain *S. xiamenensis* M1-94P.

First, we introduced a spontaneous rifampicin resistant (Rif) mutation in strain M1-94P, and we then conferred streptomycin resistance (Str) to the Rif mutant by introducing a mutated *rpsL* gene. From the HPLC fingerprints of the constructed drug resistant mutants (M1-5R22, M5, and M6), the profiles of the metabolites were found to be altered, and the production of the benzopyran compounds improved. Two novel benzopyran compounds, xiamenmycin C (**1**) and D (**2**), were isolated from the crude extracts of M6, the strain with the highest productivity of benzopyran derivatives. The structures of **1** and **2** were identified through the analysis of extensive spectroscopic data. In this study, we showed that secondary metabolite production potential in the deep-sea derivatized *Streptomyces* strain *S. xiamenensis* M1-94P was awoken by ribosomal engineering. Our results also showed that **1** and **2** have anti-fibrotic activities, and **2** is even better than xiamenmycin, due to its lower concentration and higher inhibitory effect against WI26. Our work provided unique structures for use in the study of structure-bioactivity relationships, aimed at the development of anti-fibrotic compounds for the therapeutic treatment of excessive fibrotic diseases.

## 2. Results

### 2.1. Construction of the Mutants

Based on the methodology of ribosomal engineering, the combination of rifampicin and streptomycin is the most popular pair of drugs to screen for potential high producers of bioactive compounds among drug-resistant mutants. We first screened for spontaneously occurring Rif mutants from strain M1-94P by plating the spores on a GYM (glucose 4 g/L, yeast extract 4 g/L, malt extract 10 g/L, and agar 1.5% at pH = 7.2–7.4) plate containing 10 μg/mL of rifampicin (five times minimal inhibitory concentration (MIC)). The amplified *rpoB* gene fragments from nine candidates of Rif mutation strains were sequenced to check whether the Rif mutation was targeted at RNAP or not. The mutant strain M1-5R22 was chosen for use in further strain improvements due to its altered HPLC profile and its confirmed point mutation in the *rpoB* gene (see Table 1).

A combined resistance to streptomycin in the strain M1-5R22 was obtained by introducing two types of mutated *rpsL* genes into its genome through plasmid conjugation and integration. As summarized in Table 1, all three of the mutants that showed resistance to rifampicin contained a mutation within the *rpoB* gene, in which an altered nucleotide (from G to A) was found at position 1319, which resulted in an amino acid alteration of Arg-440 to His. The generated double mutants, namely M5 and M6, contained an additional mutation in the *rpsL* gene that changed nucleotides A-262 to G or C-268 to A and T-269 to A, resulting in amino acid alterations in the ribosome protein S12 from Lys-88 to Glu or Leu-90 to Lys.

**Table 1:** Mutations in the *rpsL* and *rpoB* genes that resulted in amino acid exchanges in *S. xiamenensis* M1-94P.

Strain	Resistance *	Position in *rpoB* gene	Amino acid position (exchange)	Position in *rpsL* gene	Amino acid position (exchange)
M1-94P	-	-	-	-	-
M1-5R22	Rif	G-1319 → A	440 (Arg → His)	-	-
M5	Rif + Str	G-1319 → A	440 (Arg → His)	A-262 → G	88 (Lys → Glu)
M6	Rif + Str	G-1319 → A	440 (Arg → His)	C-268 → A T-269 → A	90 (Leu → Lys)

*Rifampicin resistance = 10 μg/mL, streptomycin resistance = 20 μg/mL.

### 2.2. HPLC Analysis of Wild-Type M1-94P and Its Mutants

**Figure 1:**
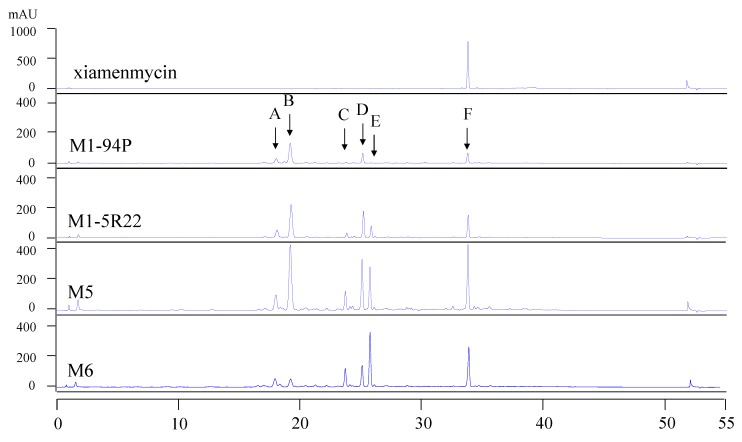
HPLC profiles of wild type M1-94P and its three mutants. The arrows (A–F) indicate the six main peaks, and arrow F indicates xiamenmycin as a standard reference.

To compare the profiles of the secondary metabolites in the wild-type M1-94P and its three mutants, HPLC analysis was performed. There are six main metabolites from M1-94P and its mutants, and these are marked by arrows A through F in the HPLC spectra (Figure 1). The peak areas of the metabolites were increased in three mutants compared with M1-94P. The double mutant strains, M5 and M6, produced several compounds (e.g., peaks A, C, and E) that were barely detected in the wild type strain M1-94P. According to the characteristics of UV absorbance and the retention time of xiamenmycin, the production of xiamenmycin, labeled as peak F, was gradually improved in the three mutants. The results suggested that mutant M6 was best suited for use in large-scale fermentations to prepare an extract containing benzopyran derivatives. Compounds **1** and **2** were isolated from the peak F with close retention time after repeatedly chromatography.

### 2.3. Structural Elucidation of Compounds **1** and **2**

**Figure 2:**
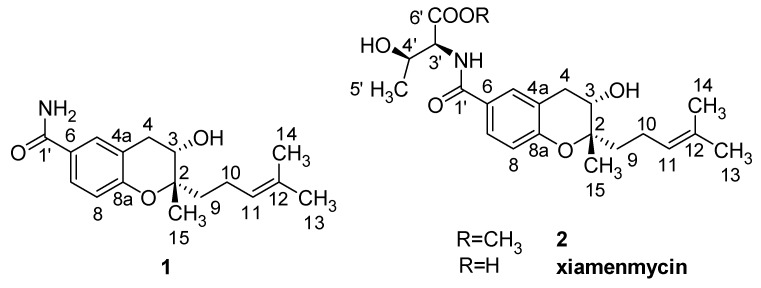
Structures of the anti-fibrotic benzopyran compounds **1**, **2**, and xiamenmycin.

The mutant M6 was selected to perform large-scale liquid fermentations under the same conditions as the small-scale fermentation. Total liquid cultures of M6 were extracted with organic solvent. Two novel benzopyran compounds **1** and **2**, as well as xiamenmycin (Figure 2), were isolated from peak F (Figure 1) after semi-preparative HPLC purification. The retention times of **1** and **2** are quite close to that of xiamenmycin during the HPLC analysis; thus, it was difficult to detect these two compounds from the crude extract initially.

The molecular formula of **1** was determined to be C_17_H_23_NO_3_ by HRESIMS (*m*/*z* = 290.1768 [M + H]^+^, positive ion mode and *m*/*z* = 288.1610 [M − H]^−^, negative ion mode). Based on similar UV characteristics, compound **1** was considered to be a derivative of xiamenmycin. The ^1^H and ^13^C NMR data, in combination with the HMBC and the HMQC correlations, resulted in the identification of benzopyran as the main structural feature. The HMBC correlations between H_2_-9 (δ_H_ 1.59, m), C-2 (δ_C_ 79.7), and C-3 (δ_C_ 66.3) confirmed the isoprenyl side chain to be located at C-2. The substituent position of the carboxamide was determined to be C-6 in the benzopyran skeleton based on the HMBC correlation between H-5 (δ_H_ 7.63, d, 1.8), H-6 (δ_H_ 7.60, dd, 8.4, 1.8) and C-1′ (δ_C_ 168). The main difference in the NMR data between **1** and xiamenmycin was found in the amino acid moieties and was due to the missing signal related to the 1′–6′ positions in the ^1^H and the ^13^C NMR spectra (Table 2). From biosynthetic reasoning and the similarity of the CD data compared with xiamenmycin, we assumed that compound **1** has the identical absolute configuration as xiamenmycin in the benzopyran portion of the molecule. Accordingly, the structure of **1** was identified as (2*S*,3*S*)-3-hydroxy-2-methyl-2-(4-methylpent-3-enyl)chroman-6-carboxamide and was named xiamenmycin C.

Compound **2** gave a *pseudo*-molecular ions [M + H]^+^ peak at *m*/*z* = 406.2210 by HRESIMS, consistent with an elemental composition of C_22_H_31_NO_6_, which was 15 amu more than the molecular weight of xiamenmycin. The ^1^H and ^13^C NMR data showed an additional methyl group due to the presence of signals at δ_H_ 3.65 (3H, s) and δ_C_ 52.3 (CH_3_). The HMBC correlations between CH_3_-7′ and CO-6′ (δ_C_ 171.8) confirmed the methyl esterification. The good agreement of the respective NMR data of **2** with those of xiamenmycin, together with ROESY data and proton coupling constants indicated that both compounds shared the same relative configurations of pyran-ring and the amino acid portion. Compound **2** possessed the same stereo-configurations as xiamenmycin, confirmed by the similar CD spectrum. Therefore, the structure of **2** was determined as (2*R*,3*S*)-methyl 3-hydroxy-2-((2*S*,3*S*)-3-hydroxy-2-methyl-2-(4-methylpent-3-enyl)chroman-6-carboxamido)butanoate, named xiamenmycin D.

**Table 2:** ^1^H and ^13^C NMR spectroscopic data of the novel benzopyran compounds **1** and **2** ^a^.

Position	1			2		
	δ_H_ (*J* in Hz)	δ_C_, type	HMBC	δ_H_ (*J* in Hz)	δ_C_, type	HMBC
1	-	-	-	-	-	-
2	-	79.7, C	-	-	79.8, C	-
3	3.74, dd (7.4, 5.2)	66.3, CH	4a, 2, 9, 15	3.76, dd (7.4, 5.2)	66.3, CH	4a, 2, 9, 15
4	2.66, dd (17.3, 7.4) 2.93, dd (17.3, 5.2)	31.3, CH_2_	8a, 5, 4a, 2, 3, 6	2.71, dd (17.3, 7.4) 2.97, dd (17.3, 5.2)	31.2, CH_2_	8a, 5, 4a, 2, 3
4a	-	120.4, C	-	-	120.6, C	-
5	7.63, d (1.8)	130.2, CH	7, 8a, 4, 1′	7.69, d (1.8)	130.0, CH	7, 8a, 4, 1′
6	-	126.3, C	-	-	125.7, C	-
7	7.60, dd (8.4, 1.8)	127.4, CH	5, 8a, 1′	7.64, dd (8.4, 1.8)	127.4, CH	5, 8a, 1′
8	6.74, d (8.4)	116.5, CH	4a, 8a, 6	6.80, d (8.4)	116.7, CH	4a, 8a, 6
8a	-	156.0, C	-	-	156.2, C	-
9	1.59, m	38.0, CH_2_	11, 12, 2, 3, 10	1.60, m	37.9, CH_2_	11, 10, 12, 2, 3
10	2.10, m	21.6, CH_2_	11, 12, 2, 9	2.11, m	21.6, CH_2_	11, 12, 2, 9
11	5.10, t (7.3)	124.8, CH	13, 10, 14, 9	5.11, t (7.2)	124.8, CH	13, 10, 14
12	-	131.3, C	-	-	131.3, C	-
13	1.56, s	18.0, CH_3_	11, 12, 14	1.57, s	17.9, CH_3_	11, 12, 14
14	1.63, s	25.9, CH_3_	11, 12, 13	1.64, s	25.9, CH_3_	11, 12, 13
15	1.16, s	18.8, CH_3_	2, 3, 9	1.18, s	18.8, CH_3_	2, 3, 9
1′	-	168.0, C	-	-	166.9, C	-
2′	-	-	-	8.02, d (8.2)	-	1′, 3′, 4′
3′	-	-	-	4.47, dd (8.1, 4.1)	59.4, CH	1′, 4′, 5′, 6′
4′	-	-	-	4.17, dq (6.3, 4.1)	66.9, CH	5′, 6′
5′	-	-	-	1.14, d (6.3)	20.7, CH_3_	3′, 4′
6′	-	-	-	-	171.8, C	-
7′	-	-	-	3.65, s	52.3, CH_3_	6′
CO–NH_2_	8.38, brs	-	-	-	-	-

^a^ Measured in DMSO-*d*_6_, chemical shifts (δ) in ppm.

### 2.4. Inhibition of the Proliferation of Human Diploid Lung Fibroblast (WI26) Cells by Compounds **1** and **2**

**Figure 3:**
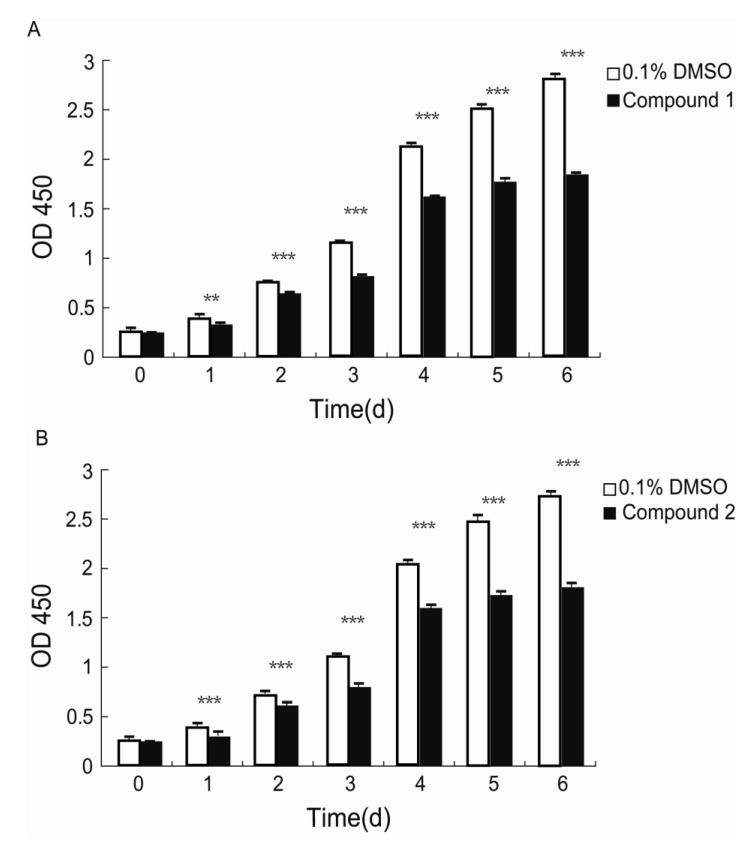
Inhibitory effects of compounds **1** and **2** on the proliferation of WI26 cells. The WI26 cells were exposed to 15 μg/mL of **1** and 30 μg/mL of **2** at day 0, 1, 2, 3, 4, 5 and 6. The surviving fraction was determined by Cell Counting Kit-8 assay. As illustrated below, the proliferation of the WI26 cells was significantly inhibited by **1** (**A**) and **2** (**B**) in a time-dependent manner. The data are given as the means of triplicate values ± SD of three independent experiments. Significant differences from the value of the control sample treated with only 0.1% DMSO solvent are marked. ** *p* < 0.01, *** *p* < 0.001.

Fibroblasts play pivotal roles in establishing and maintaining the self-perpetuating inflammatory circuits and are, in fact, the main ECM generating cells in fibrotic diseases [18]. Thus, finding a small molecule that can slow the rapid fibrotic response would be a reasonable way to develop an anti-fibrotic drug. We investigated the anti-proliferative activities of **1** and **2** against human lung fibroblast. WI26 cells were exposed to **1** at a concentration of 15 μg/mL, **2** at a concentration of 30 μg/mL, and xiamenmycin at a concentration of 30 μg/mL, respectively, for 0, 1, 2, 3, 4, 5, and 6 days, and the optical density (OD) at λ = 450 nm was monitored as a function of time. As illustrated in Figure 3, compound **1** significantly inhibited the proliferation of the WI26 cells at lower concentration, compared with both **2** and xiamenmycin. The extent of this inhibition of **1** increased with time, from a decrease of 13.8% at day one to a decrease of 38% at day six at a concentration of 15 μg/mL, compared with a control sample that was only treated with solvent (Figure 3A, Table S1). Therefore, compound **1** does indeed exhibit better anti-proliferative effects on human lung fibroblast. Compared with xiamenmycin, **2** showed similar anti-fibrotic bioactivity, which can inhibit the proliferation from a decrease of 12.8% at day one to a decrease of 38% at day six at a concentration of 30 μg/mL (Figure 3B, Table S1).

## 3. Discussion

The wild-type *S. xiamenensis* strain M1-94P, an affined strain of *S. xiamenensis* 318, was isolated from deep-sea sediments and used as a biosource in the search for new benzopyran compounds. The possibility of discovering novel chemical structures may be higher when shifting to an alien niche, such as deep-sea derived microbial strains, from the view of chemical ecology, but the production of secondary metabolites in the deep-sea derived microbes is normally quite low, due to physiological stress, when they are cultivated under the usual laboratory conditions. Therefore, the use of a rational strain development approach such as ribosomal engineering may be useful to increase the production of secondary metabolites in the laboratory.

Improving the production of the secondary metabolites by bacteria by modulating the ribosome components as well as other translating factors or RNAP [15] has several advantages, including the ability to screen for drug-resistant mutations by simple selection on drug-containing plates and without prior genetic mutagenesis [19]. It was reported that the introduction of multiple drug resistant mutations had the cumulative effect of increasing the production of secondary metabolites, which led to a dramatic increase in the production of the bioactive compounds. Rifampicin and streptomycin is a pair of drugs widely used in ribosomal engineering to screen potential high producers among drug-resistant mutants. In our case, the introduction of combined drug resistance to rifampicin and streptomycin in *S. xiamenensis* M1-94P was a successful method to improve the production of benzopyran compounds for chemical isolation, although the detailed mechanism of the enhancement of the specific natural product compound remains unknown.

Because spontaneous rifampicin resistance mutants always have point mutations clustered in the so-called Rif domain on the *rpoB* gene (encoding the β-subunit of RNA polymerase) and could mimic the ppGpp binding effect on the mutated RNAP [20], we decided to screen for a Rif resistant mutant as the first step in the strain development. We have sequenced the *rpoB* gene fragment of nine Rif mutants including six Rif mutants from 5 × MIC and 3 Rif mutants from 10 × MIC. All of the mutants have a point mutation in the Rif domain in the *rpoB* gene.

There are two types of streptomycin resistance that could effectively alter ribosomal function. Determination of the mutation in the ribosomal RNA that engenders a low level of streptomycin resistance is not easy, but the introduction of a mutation into the highly conserved ribosomal protein S12 is conveniently achieved by site-directed mutagenesis using PCR (polymerase chain reaction). Furthermore, certain *rpsL* mutations, resulting in the amino acid substitution of either K88E or L90K, that confer resistance to Str can increase the production of secondary metabolites in *Streptomyces* [21,22,23,24,25]. Thus, it is more rational to engineer ribosomal protein S12 as the second step in the strain development; thus, we introduced a mutated *rpsL* gene to confer streptomycin resistance to the Rif mutant M1-5R22 and to interfere with its ribosomal function. As shown in the HPLC profiles, the production of the target compounds, xiamenmycin increased drastically in the Rif-Str double resistance strains M5 and M6.

Xiamenmycin, xiamenmycin C (**1**) and D (**2**), with highly similar chemical structures, were all isolated from mutant M6 of deep-sea derived *S. xiamenensis* M1-94P. Compared with xiamenmycin, obtained from our previous study [11], xiamenmycin C had the same benzopyran skeleton and isoprene side chain. The only difference in the structures is in the amino acid moiety (on position 1′). Compound **1** is the possible precursor of xiamenmycin during biosynthesis. Compound **2** is methyl ester of xiamenmycin, which may be synthesized in the tailing step of the biosynthetic pathway. From the view of combinatorial biosynthesis, more “unnatural” natural products may be produced by the elucidation of the biosynthetic pathway for the synthesis of benzopyran compounds.

Xiamenmycin C (**1**) was found to exhibit better inhibitory effects on the cell proliferation of human lung fibroblasts (WI26) using lower doses compared with xiamenmycin. It seems that the benzopyran skeleton is crucial for the anti-fibrotic activity. Xiamenmycin is an antifibrotic small molecule that targets the inflammatory and mechanical stress responses, the two pivotal pathological processes that occur during excessive fibrogenesis [12]. Xiamenmycin D (**2**) showed similar anti-fibrotic activity, compared with xiamenmycin. It indicated the methylation on position 6′ may not affect the bioactivity significantly. As a type of potential anti-fibrotic drug, more diverse benzopyran structures are needed for the investigation of the structure-activity relationship (SAR). For example, compounds with distinct substituent groups on the benzopyran skeleton are expected from natural sources, or a variation in the benzopyran configuration could be achieved by total or partial synthesis.

In summary, our results showed that xiamenmycin C and D, two new benzopyran structures, can be isolated from the deep-sea derived *S. xiamenensis* M1-94P. Ribosomal engineering, *i.e.*, the introduction of a spontaneous rifampicin resistance mutation combined with the introduction of a streptomycin resistant mutation, was performed to generate mutant M6 with enhanced secondary metabolite production. As a promising candidate for treating excessive fibrotic diseases, the effects of xiamenmycin and its derivatives on mechanical stress and inflammation in association with various fibroblast cellular behaviors will be examined in the next stage of research.

## 4. Experimental Section

### 4.1. General

^1^H and ^13^C NMR spectra were recorded with Bruker DRX-500 and Advance Ⅲ-600 NMR spectrometers using the solvent as an internal standard (DMSO*-d_6_* δ = 2.51 and 40.0 ppm, respectively). Coupling constants (*J*) are reported in Hertz (Hz) and chemical shifts (δ) are expressed in parts per million (ppm). Optical rotation was measured by a JASCO P-2000 polarimeter, and CD spectra were recorded on a J-815 spectropolarimeter (JASCO, Gross-Umstadt, Germany) at room temperature. UPLC-HRMS was measured on a Waters ACQUITY UPLC system equipped with a binary solvent delivery manager and a sample manager coupled with a Waters Micromass Q-TOF Premier Mass Spectrometer equipped with an electrospray interface (Waters Corporation, Milford, MA, USA). HPLC was performed on Agilent Technologies 1200 series instrument (Agilent Technologies, Wadbronn, Germany). Column chromatography was carried out with Sephadex LH-20 (40–70 μm, Amersham Pharmacia Biotech AB, Uppsala, Sweden), silica gel (200–300 mesh, Qingdao Marine Chemical, Inc., Qingdao, China), Lichroprep reversed-phase RP-18 silica gel (40–63 μm, Merck, Darmstadt, Germany) and silica gel H (10–40 μm, Qingdao, China). Analytical HPLC was carried out on an Agilent XDB-C18 column (4.6 × 150 mm, 5 μm) with a flow rate of 1 mL/min. Organic solvents for HPLC were analytical grade and were purchased from Merck KGaA (Darmstadt, Germany).

### 4.2. The Original Strain Materials

The *Streptomyces* xiamenensis strain M1-94P was isolated from a deep-sea sediment sample collected at the depth of 2628 m in the Eastern Pacific (12.7115′N, 103.9071′W). This strain M1-94P was determined to be *S. xiamenensis* by 16S rRNA gene sequence analysis.

### 4.3. Construction of Mutants by the Screening for Spontaneous Rifampicin Resistance

Fresh spores of wild strain M1-94P formed by cultivation on SFM (soy flour 20 g/L, d-mannitol 20 g/L, agar 1.5%, pH = 7.2) plates were incubated at 30 °C for 7 days and were then harvested and suspended in the appropriate amount of sterilized and distilled water. They were filtered to remove medium fragments and were then preserved in the 20% (v/v) glycerol tube (2 mL).

The M1-94P spore suspension was spread on GYM agar medium (glucose 4 g/L, yeast extract 4 g/L, malt extract 4 g/L, and agar 1.5% at pH = 7.2–7.4) containing various rifampicin concentrations. The MIC of rifampicin against M1-94P was determined through a two day incubation period at 30 °C on GYM agar medium. Spontaneous Rif mutants were obtained from the colonies that grew within 7–10 days after the spores of M1-94P were spread on the GYM agar medium containing different rifampicin concentrations (5 × MIC, 10 × MIC, 50 × MIC). Spontaneous drug resistant mutants were selected for further characterization.

### 4.4. Introduction of Streptomycin Resistance by Site Directed Mutagenesis

The *rpsL* gene fragment was amplified by PCR using the M1-94P genomic DNA as the template and was subjected to site-directed mutagenesis using PCR. The sequences of the forward primers were (mutagenic positions underlined): K88E-F (5′-GGCCGTGTGGAGGACCTGCCGGGTG-3′) and L90K-F (5′-GTGTGAAGGACAAGCCGGGTGTCCG-3′). The utilized reverse primers, K88E-R and L90K-R, were complementary to the forward primers. Primer pairs XJ1F (5′-CATATGGTGCCAACGATCCAGCA-3′) and XJ1R (5′-GATATCTTACTTCTCCTTCTTGGCGC-3′) were designed to introduce *Nde*I and *Eco*RV sites (underlined below) at the translation start and stop codon of the *rpsL* gene and combined with the primers K88E-F/R and L90K-F/R to generate point mutations in the mutagenesis PCR amplification.

The entire length of the mutated *rpsL* gene was amplified by PCR and inserted into the pMD 18-T vector to check for site directed mutagenesis by sequencing, which generated plasmids p822 (K88E) and p827 (L90K). The *Nde*I-*Eco*RV fragments containing mutated *rpsL* genes were excised and inserted into the same sites of pIB139, an integrative expression vector in *Streptomyces* [26], generating pIB139-822 (K88E) and pIB139-827 (L90K). These two plasmids were transformed into *E. coli* ET12567: pUZ8002 separately and used as the donor strains for two parental *E. coli*—*Streptomyces* conjugations. Exoconjugants derived from the wild type *S. xiamenensis* M1-5R22 were selected by rifampicin and apramycin on the SFM solid medium plates [27]. The mutants harboring pIB139-822 and pIB139-827 were named M5 and M6, respectively.

### 4.5. Mutation Analysis of the *rpsL* and *rpoB* Genes

The primers for the PCR amplification of the *rpoB* and the *rpsL* genes were designed using the sequence information of the draft genome of *S. xiamenensis* (unpublished data). The partial *rpoB* gene fragments (nucleotides 374–1582, 1.2 kb) of the wild strain M1-94P and its Rif-resistant mutants (M1-5R22, M5 and M6) were obtained by PCR using their genomic DNA as templates and the synthetic oligonucleotide primers (forward: 5′-CCGAGTTCACCAACAACGAGACC-3′, reverse: 5′-CGATGACGAAGCGGTCCTCC-3′). The complete *rpsL* gene was amplified from the wild strain M1-94P and its drug-resistant mutants (M1-5R22, M5 and M6) by primer pairs (forward: 5′-TGTCCTCGGGTATCGGTCTG-3′ and reverse: 5′-TTACTTCTCCTTCTTGGCGCCGTAG-3′). The PCR products were directly sequenced by Sangon Biotech (Shanghai, China) Co., Ltd.

### 4.6. HPLC Analysis

Spore suspensions of the wild type strain M1-94P and its three mutants were inoculated in test tubes (15 × 150 mm) with 5 mL of Tryptone Soy Broth (TSB) (Oxoid, Hampshire, UK) and were pre-cultured at 30 °C for 1 day on a rotary shaker at 280 rpm. The TSB broth was then transferred into a 500 mL flask containing 100 mL of yeast extract-malt extract broth (GYM medium) and was cultured at 30 °C for 7 days on a rotary shaker at 280 rpm. Each culture was centrifuged at 9000 rpm for 10 min, and the supernatant was then extracted three times at room temperature overnight with equal volumes of the solvent ethyl acetate. The supernatant was combined and concentrated under vacuum at 37 °C to remove the organic phase. Each crude extract was subsequently dissolved in the same volume of HPLC grade methanol.

The samples were analyzed by HPLC using an Agilent XDB-C18 column (4.6 × 150 mm, 5 μm) and monitored by UV detection at 254 nm. The solvent system of methanol (A) and H_2_O (B) was used as the mobile phase in the following linear gradient: 0 min 10% B, 10 min 10% B, 40 min 100% B, 50 min 100% B, 51 min 10% B, 55 min 10% B, at a flow rate of 1 mL/min. The xiamenmycin obtained by our group was used as the standard reference.

### 4.7. Fermentation, Extraction, and Isolation

Mutant M6 was selected to perform a large-scale fermentation (33 L) in yeast extract-malt extract broth. The liquid culture was centrifuged at 9000 rpm for 10 min, and the supernatant was extracted at room temperature by ethyl acetate. The residue was extracted at room temperature overnight by a solvent mixture of ethyl acetate:methanol:acetic acid (80:5:5, v:v:v). The second supernatant was then filtered, and the residue was extracted twice more as described above. All the supernatants were combined and concentrated under vacuum at 37 °C to remove the organic phase. The crude extract (13.4 g) was obtained and was then subjected to silica gel column chromatography with elution by a mixture of dichloromethane (CH_2_Cl_2_) and methanol (MeOH) (gradient from CH_2_Cl_2_ (300 mL), 70:1 (v:v, 300 mL), 60:1 (v:v, 300 mL), 50:1 (v:v, 1.5 L), 30:1 (v:v, 800 mL), 10:1 (v:v, 1.5 L), 5:1 (v:v, 1 L), 2:1 (v:v, 1 L), 1:1 (v:v, 600 mL) to MeOH (600 mL)) and ten fractions (Fr.A–Fr.J) were obtained. Guided by HPLC fingerprinting, two fractions, Fr.F (3.09 g) eluted by CH_2_Cl_2_:MeOH = 15:1 to 10:1 and Fr.H (1.17 g) eluted by CH_2_Cl_2_:MeOH = 2:1, were collected and further subjected to Sephadex LH-20 column chromatography with elution by 100% methanol.

Fr.F-2 (0.478 g) was a subfraction of Fr.F, which was identified as the target fraction according to the HPLC fingerprints. Fr.F-2 was subsequently subjected to silica gel column chromatography and was eluted with CH_2_Cl_2_:MeOH. Fr.F-2-4 (eluted with CH_2_Cl_2_:MeOH (40:1, v:v)) showed the target peaks of the xiamenmycin derivatives from UV characterization at λ_max_ = 206 and 260 nm. Fr.F-2-4 (18 mg) was further purified again by semi-preparative HPLC (Agilent ZOBRAX-C18 column, 5 μm, 9.4 × 250 mm), with the following gradient: CH_3_CN (A)/H_2_O (B): 0 min 42% A, 8 min 42% A, 33 min 72% A, 34 min 100% A, 50 min 100% A, at a flow rate of 1.5 mL/min. One sub-fraction Fr.F-2-4-2 contained compound **2** (1.5 mg). Fr.F-2-2 (8 mg) was subsequently purified by semi-preparative HPLC (Agilent ZOBRAX-C18 column, 5 μm, 9.4 × 250 mm), with the following gradient: CH_3_CN (A)/H_2_O (B): 0 min 45% A, 5 min 45% A, 35 min 55% A, 36 min 100% A, 50 min 100% A, at the flow rate of 1.5 mL/min. One sub fraction Fr.F-2-2-3 was collected as compound **1** (1.5 mg).

Compound **1**: Yellow amorphous powder (MeOH); [α]^26^_D_ +28.45° (*c* 0.0034, MeOH); UV (MeOH) λ_max_ = 206, 260 nm; CD (*c* 0.0024, MeOH) Δε_201_ +12.3, Δε_202_ +9.6, Δε_205_ +8.0, Δε_207.4_ +7.1, Δε_213.6_ +0.14, Δε_217_ −1.2, Δε_245.2_ +2.0, Δε_259.8_ +3.14, Δε_283_ +0.03; ^1^H and ^13^C NMR data, see Table 2; HRESIMS *m*/*z* 290.1768 [M + H]^+^, (calcd. for C_17_H_24_NO_3_, *m*/*z* 290.1756), 288.1610 [M − H]^−^, (calcd. for C_17_H_22_NO_3_, *m*/*z* 288.2073).

Compound **2**: Yellow amorphous powder (MeOH); [α]^30^_D_ +6.52° (*c* 0.0024, MeOH); UV (MeOH) λ_max_ 206, 260 nm; CD (*c* 0.0034, MeOH) Δε_205.6_ +6.7, Δε_206.9_ +5.5, Δε_213_ +20.1, Δε_223.4_ +0.85, Δε_260_ +9.23, Δε_296.8_ +1.0; ^1^H and ^13^C NMR data, see Table 2; HRESIMS *m*/*z* 406.2210 [M + H]^+^, (cacld. for C_22_H_32_NO_6_, *m*/*z* 406.2230).

### 4.8. Cell Proliferation Assay

The effects of compounds **1** and **2**, as well as xiamenmycin on cell proliferation were determined using a standard Cell Counting Kit-8 (CCK-8, Dojindo, Kumamoto, Japan) assay according to the manufacturers instructions. WI26 cells at 70%–80% confluency were treated with a 0.25% trypsin and 0.02% EDTA solution, centrifuged and resuspended in DMEM supplemented with 10% FBS and antibiotics. The cells were then seeded in 96-well plates (100 μL/well) at an initial density of 2.5 × 10^4^ cells/mL. The medium was replaced 24 h later by fresh DMEM with 10% FBS and antibiotics containing 15 μg/mL of compound **1**, 30 μg/mL of compound **2**, 30 μg/mL of xiamenmycin, or 0.1% DMSO (AppliChem, Darmstadt, Germany). Subsequently, the medium was refreshed and the viabilities of the cells were measured by using a CCK-8 solution at day 0, 1, 2, 3, 4, 5, and 6, respectively. A proliferation measurement was performed by adding 10 μL of CCK-8 solution to each well and incubating the solutions at 37 °C for 1 h. The OD values of each well were measured at the primary wavelength λ = 450 nm using a Microplate Spectrophotometer (PowerWaveXS, BioTek, Seattle, WA, USA). The data are shown as the means ± standard deviations (SD) of three independent experiments, each performed in triplicate.

### 4.9. Statistical Analysis

Statistical differences were calculated using student’s paired *t*-test at significance levels of *p* < 0.05 to 0.001.

## 5. Conclusions

The benzopyran compound xiamenmycin can be obtained through the cultivation of a mangrove-derived strain of *Streptomyces xiamenensis*, strain 318, and it has multiple anti-fibrotic effects. The deep sea*-*derived *S. xiamenensis* strain M1-94P was selected for further chemical and pharmaceutical studies of benzopyran compounds. The introduction of spontaneous rifampicin resistance combined with streptomycin resistance by introducing a mutated *rpsL* gene into M1-94P was performed to increase the production of its secondary metabolites. Two new benzopyran derivatives, xiamenmycin C (**1**) and D (**2**), were isolated from one of the Rif-Str double mutants, M6. The structures of **1** and **2** were identified through extensive spectroscopic data analysis. The isolation of the novel compounds increased the structural diversity of known benzopyran compounds, and the anti-proliferation bioactivities of **1** and **2** on human lung fibroblast behavior were investigated. Compared with xiamenmycin, the bioactivity of **1** is increased when the amino acid moiety was removed and compound **2** showed similar anti-fibrotic activity. Our work may be useful for a structure-activity relationship study of anti-fibrotic benzopyran compounds to develop leading drugs for medical treatment of excessive fibrotic disease from marine natural product.

## Supplementary Files

Supplementary File 1 Supplementary Materials (PDF, 1338 KB)

## References

[1] Vlietinck A.J., de Bruyne T., Apers S., Pieters L.A. (1998). Plant-derived leading compounds for chemotherapy of human immunodeficiency virus (HIV) infection. Planta Med..

[2] Borm P., Greim H., Snyder R. (2008). Toxicity of Selected: Toxicology of Fibers and Particles. Proceedings of Toxicology and Risk Assessment.

[3] Wynn T.A. (2008). Cellular and molecular mechanisms of fibrosis. J. Pathol..

[4] Friedman S.L., Sheppard D., Duffield J.S., Violette S. (2013). Therapy for fibrotic diseases: Nearing the starting line. Sci. Transl. Med..

[5] Wynn T.A., Ramalingam T.R. (2012). Mechanisms of fibrosis: Therapeutic translation for fibrotic disease. Nat. Med..

[6] Machado N.F.L., Marques M.P.M. (2010). Bioactive chromone derivatives—Structural diversity. Curr. Bioact. Compd..

[7] Evans J.M., Fake C.S., Hamilton T.C., Poyser R.H., Watts E.A. (1983). Synthesis and antihypertensive activity of substituted *trans*-4-amino-3,4-dihydro-2,2-dimethyl-2*H*-1-benzopyran-3-ols. J. Med. Chem..

[8] Göker H., Boykin D.W., Yıldız S. (2005). Synthesis and potent antimicrobial activity of some novel 2-phenyl or methyl-4*H*-1-benzopyran-4-ones carrying amidinobenzimidazoles. Bioorg. Med.Chem..

[9] Kawamura N., Tsuji E., Watanabe Y., Tsuchihashi K., Takako T. (2000). Benzopyran Derivatives, Their Manufacture with *Streptomyces* Species, and Their Use for Treatment of Asthma and Rheumatoid Arthritis. Jpn. Patent.

[10] Xu J., Wang Y., Xie S.J., Xiao J., Ruan J.S. (2009). *Streptomyces xiamenensis* sp. nov., isolated from mangrove sediment. Int. J. Syst. Evol. Microbiol..

[11] Xu M.J., Liu X.J., Zhao Y.L., Liu D., Xu Z.H., Lang X.M., Ao P., Lin W.H., Yang S.L., Zhang Z.G. (2012). Identification and characterization of an anti-fibrotic benzopyran compound isolated from mangrove-derived *Streptomyces xiamenensis*. Mar. Drugs.

[12] Liu X.J., Xu M.J., Fan S.T., Wu Z., Li J., Yang X.M., Wang Y.H., Xu J., Zhang Z.G. (2013). Xiamenmycin attenuates hypertrophic scars by suppressing local inflammation and the effects of mechanical stress. J. Invest. Dermatol..

[13] Nathan C., Ding A.H. (2010). Nonresolving inflammation. Cell.

[14] Ochi K. (2007). From microbial differentiation to ribosome engineering. Biosci. Biotechnol. Biochem..

[15] Ochi K., Okamoto S., Tozawa Y., Inaoka T., Hosaka T., Xu J., Kurosawa K. (2004). Ribosome engineering and secondary metabolite production. Adv. Appl. Microbiol..

[16] Tanaka Y., Kasahara K., Hirose Y., Murakami K., Kugimiya R., Ochi K. (2013). Activation and products of the cryptic secondary metabolite biosynthetic gene clusters by rifampin resistance (rpoB) mutations in actinomycetes. J. Bacteriol..

[17] Ochi K., Hosaka T. (2013). New strategies for drug discovery: Activation of silent or weakly expressed microbial gene clusters. Appl. Microbiol. Biotechnol..

[18] Eckes B., Nischt R., Krieg T. (2010). Cell-matrix interactions in dermal repair and scarring. Fibrogenesis Tissue Repair.

[19] Wang G., Hosaka T., Ochi K. (2008). Dramatic activation of antibiotic production in *Streptomyces coelicolor* by cumulative drug resistance mutations. Appl. Environ. Microbiol..

[20] Xu J., Tozawa Y., Lai C., Hayashi H., Ochi K. (2002). A rifampicin resistance mutation in the *rpoB* gene confers ppGpp-independent antibiotic production in *Streptomyces coelicolor* A3(2). Mol. Genet. Genomics.

[21] Okamoto-Hosoya Y., Okamoto S., Ochi K. (2003). Development of antibiotic-overproducing strains by site-directed mutagenesis of the *rpsL* gene in *Streptomyces lividans*. Appl. Environ. Microbiol..

[22] Hu H., Ochi K. (2001). Novel approach for improving the productivity of antibiotic-producing strains by inducing combined resistant mutations. Appl. Environ. Microbiol..

[23] Hesketh A., Ochi K. (1997). A novel method for improving *Streptomyces coelicolor* A3(2) for production of actinorhodin by introduction of *rpsL* (encoding ribosomal protein S12) mutations conferring resistance to streptomycin. J. Antibiot. (Tokyo).

[24] Okamoto-Hosoya Y., Sato T.A., Ochi K. (2000). Resistance to paromomycin is conferred by *rpsL* mutations, accompanied by an enhanced antibiotic production in *Streptomyces coelicolor* A3(2). J. Antibiot. (Tokyo).

[25] Shima J., Hesketh A., Okamoto S., Kawamoto S., Ochi K. (1996). Induction of actinorhodin production by *rpsL* (encoding ribosomal protein S12) mutations that confer streptomycin resistance in *Streptomyces lividans* and *Streptomyces coelicolor* A3(2). J. Bacteriol..

[26] Wilkinson C.J., Hughes-Thomas Z.A., Martin C.J., Bohm I., Mironenko T., Deacon M., Wheatcroft M., Wirtz G., Staunton J., Leadlay P.F. (2002). Increasing the efficiency of heterologous promoters in actinomycetes. J. Mol. Microbiol. Biotechnol..

[27] Kieser T., Bibb M.J., Buttner M.J., Chater K.F., Hopwood D.A. (2000). Practical Streptomyces Genetics.

