# Supplementary material for: Identification of Two Novel Anti-Fibrotic Benzopyran Compounds Produced by Engineered Strains Derived from Streptomyces xiamenensis M1-94P that Originated from Deep-Sea Sediments
Source: Mar Drugs. 2013 Oct 22;11(10):4035–49. doi: 10.3390/md11104035 (PMC3826148; doi:10.3390/md11104035)

Supplementary Materials

Table S1. Anti-proliferation effect on WI26 of compounds **1**, **2** and xiamenmycin 2
Figure S1. HRESIMS spectra of compound **1** (positive and negative modes)............................... 2
Figure S2. UV spectra of compound **1** (online detection by HPLC)... 3
Figure S3. ^{1}H NMR spectrum of compound **1** in DMSO-d_6. 3
Figure S4. ^{13}C NMR spectrum of compound **1** in DMSO-d_6 4
Figure S5. HMBC spectrum of compound **1** in DMSO-d_6... 4
Figure S6. HSQC spectrum of compound **1** in DMSO-d_6.. 5
Figure S7. COSY spectrum of compound **1** in DMSO-d_6.. 5
Figure S8. NOSEY spectrum of compound **1** 6
Figure S9. CD spectrum of compound **1** 6
Figure S10. HRESIMS spectra of compound **2** (positive mode) 7
Figure S11. UV spectra of compound **2** (online detection by HPLC).. 7
Figure S12. ^{1}H NMR spectrum of compound **2** in DMSO-d_6 8
Figure S13. ^{13}C NMR spectrum of compound **2** in DMSO-d_6 8
Figure S14. HMBC spectrum of compound **2** in DMSO-d_6.. 9
Figure S15. HSQC spectrum of compound **2** in DMSO-d_6... 9
Figure S16. COSY spectrum of compound **2** in DMSO-d_6... 10
Figure S17. ROSEY spectrum of compound **2** in DMSO-d_6 10
Figure S18. CD spectrum of compound **2** 11

Table S1. Anti-proliferation effect on WI26 of compounds **1**, **2** and xiamenmycin.

	**Compound 1 (15 μg/mL)**	**Compound 2 (30 μg/mL)**	**Xiamenmycin (30 μg/mL)**
1 day	13.8%	12.8%	10%
2 day	18.2%	15%	14.9%
3 day	28.6%	27.8%	18.9%
4 day	30%	28.5%	20%
5 day	33.7%	30.3%	25.4%
6 day	38%	31.2%	28.5%

Figure S1. HRESIMS spectra of compound **1**.

Elemental Composition Report **Page 1**

Single Mass Analysis
Tolerance = 10.0 mDa / DBE: min = -1.5, max = 50.0
Element prediction: Off
Number of isotope peaks used for i-FIT = 3

Monoisotopic Mass, Even Electron Ions
144 formula(e) evaluated with 1 results within limits (up to 50 closest results for each mass)
Elements Used:
C: 0-30 H: 0-50 N: 0-2 O: 0-20
2-4-2
XX_Y05775_03 379 (8.480) Cm (379) 1: TOF MS ES+
3.98e+004

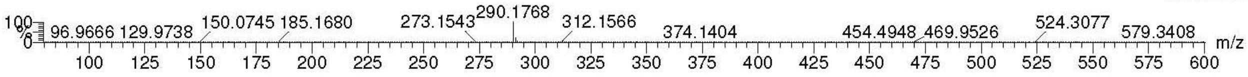

Minimum:				-1.5				
Maximum:		10.0	100.0	50.0				
Mass	Calc. Mass	mDa	PPM	DBE	i-FIT	Norm	Conf(%)	Formula
290.1768	290.1756	1.2	4.1	6.5	33.3	n/a	n/a	C17 H24 N O3

Elemental Composition Report **Page 1**

Single Mass Analysis
Tolerance = 10.0 mDa / DBE: min = -1.5, max = 50.0
Element prediction: Off
Number of isotope peaks used for i-FIT = 3

Monoisotopic Mass, Odd and Even Electron Ions
144 formula(e) evaluated with 7 results within limits (up to 50 closest results for each mass)
Elements Used:
C: 0-30 H: 0-50 N: 0-2 O: 0-20
Fr.F-2-4-Z-F-2-4-2
2013031222 668 (3.866) Cm (667:668) 1: TOF MSMS 288.19ES-
5.45e+002

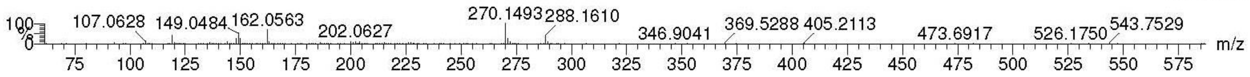

Minimum:				-1.5				
Maximum:		10.0	100.0	50.0				
Mass	Calc. Mass	mDa	PPM	DBE	i-FIT	Norm	Conf(%)	Formula
288.1610	288.1600	1.0	3.5	7.5	15.2	1.702	18.23	C17 H22 N O3
	288.1626	-1.6	-5.6	12.0	14.7	1.234	29.11	C20 H20 N2
	288.1573	3.7	12.8	3.0	16.0	2.508	8.14	C14 H24 O6
	288.1658	-4.8	-16.7	-1.5	17.1	3.641	2.62	C10 H26 N O8
	288.1685	-7.5	-26.0	3.0	16.3	2.789	6.15	C13 H24 N2 O5
	288.1533	7.7	26.7	-1.0	17.3	3.856	2.12	C9 H24 N2 O8
	288.1514	9.6	33.3	12.0	14.6	1.090	33.63	C21 H20 O

Figure S2. UV spectra of compound **1** (online detection by HPLC).

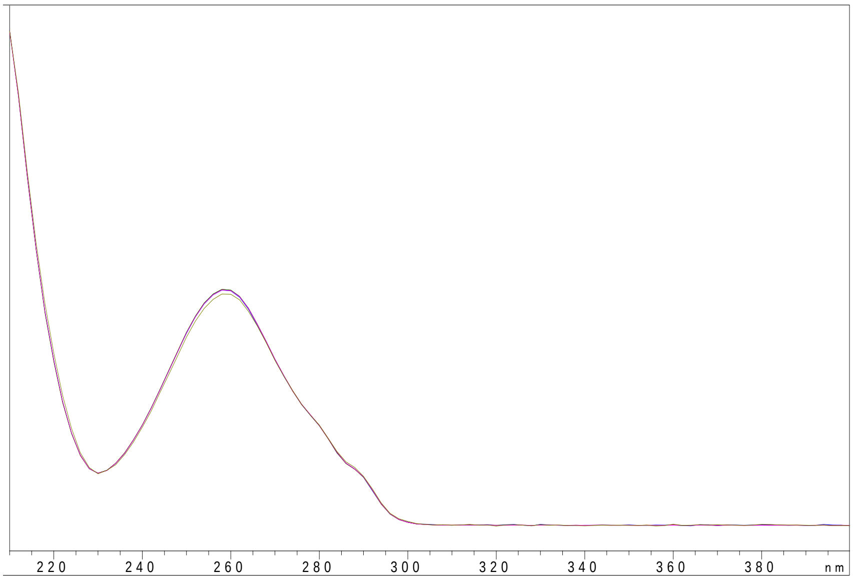

Figure S3. ^{1}H NMR spectrum of compound **1** in DMSO-d_6.

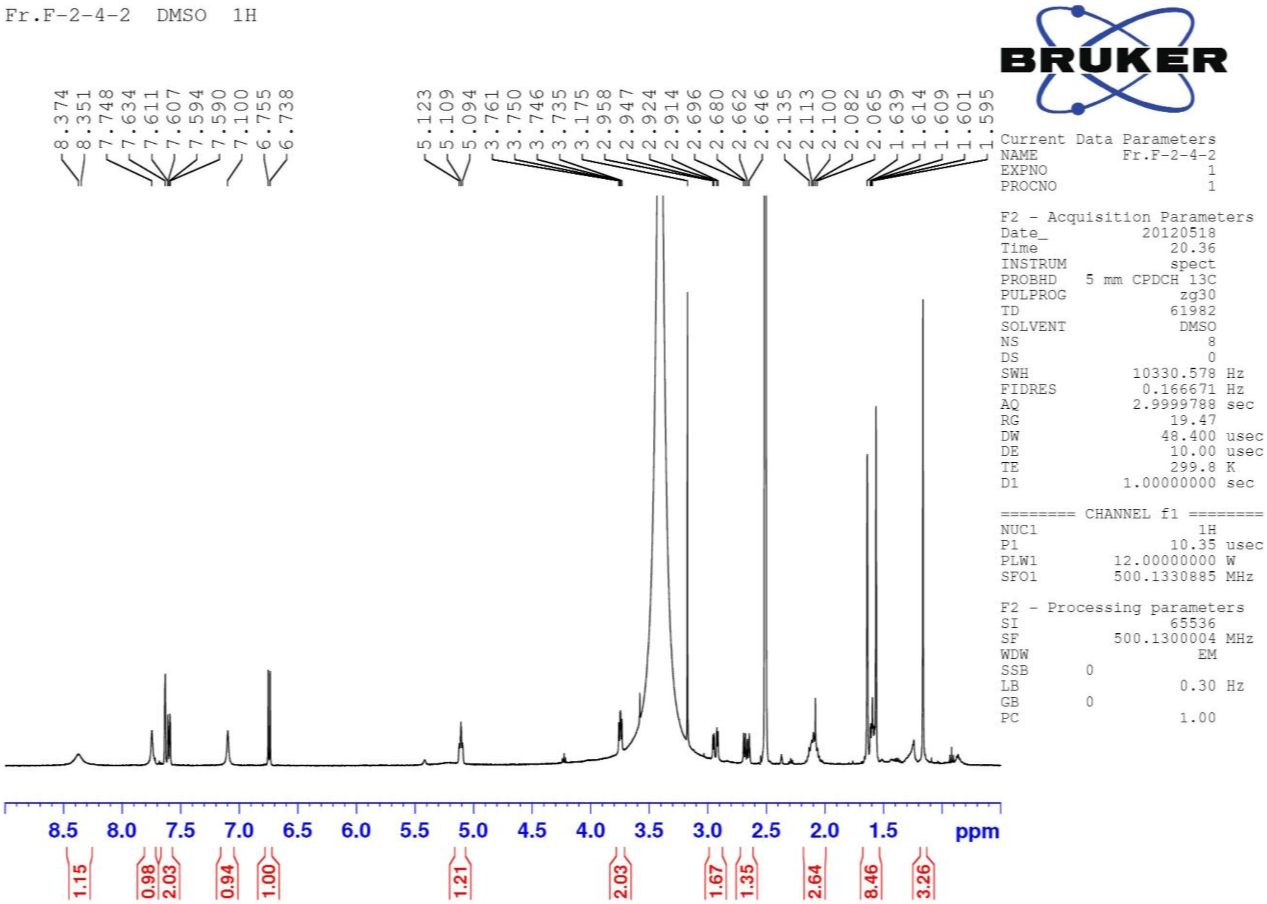

Figure S4. ^{13}C NMR spectrum of compound **1** in DMSO-d_6.

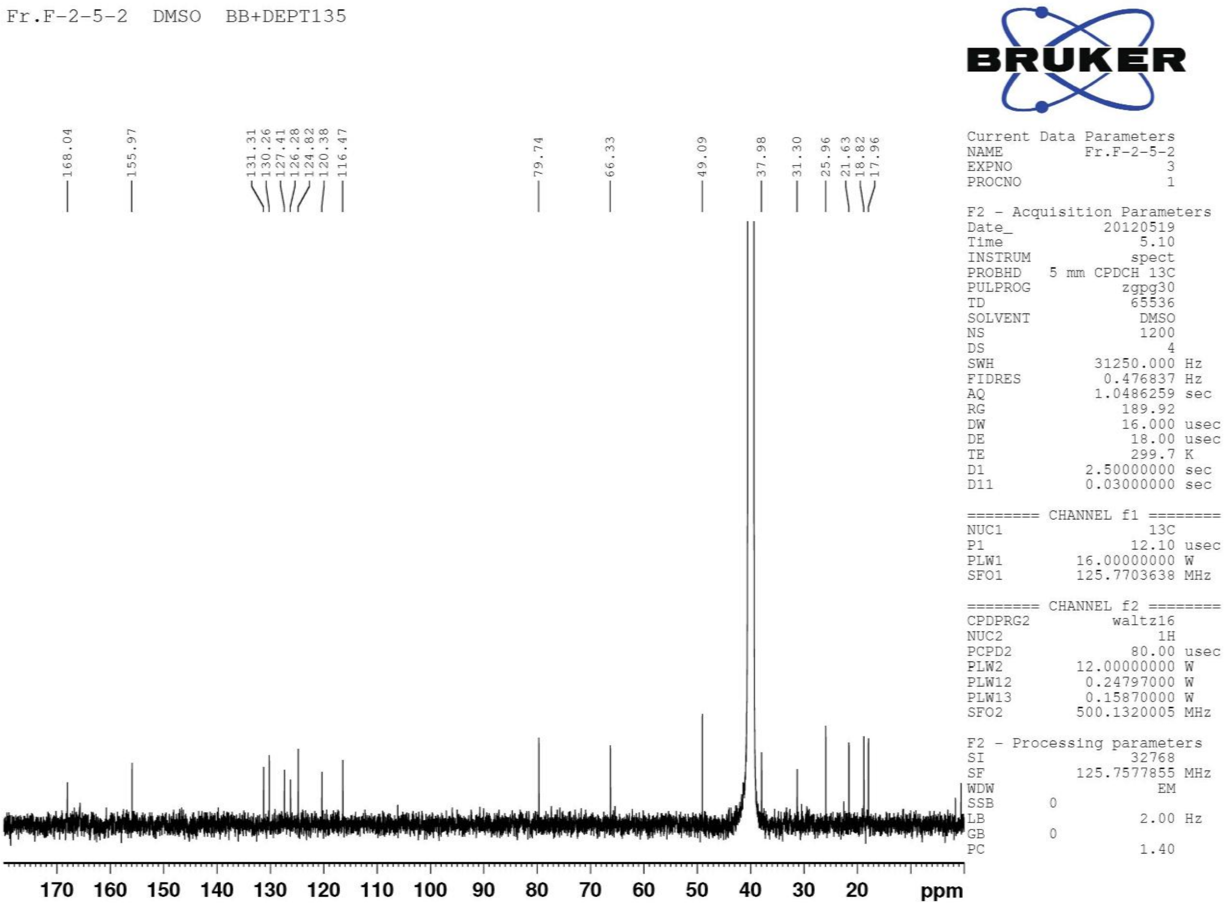

Figure S5. HMBC spectrum of compound **1** in DMSO-d_6.

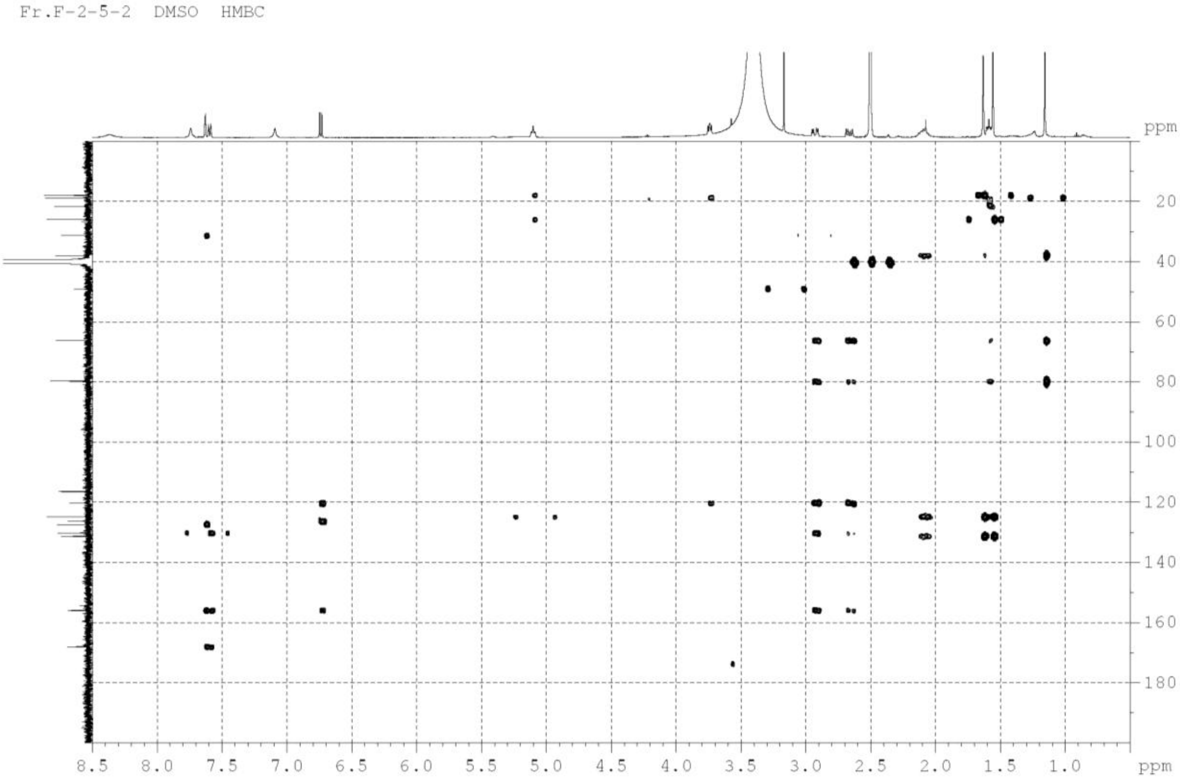

Figure S6. HSQC spectrum of compound **1** in DMSO-d_6.

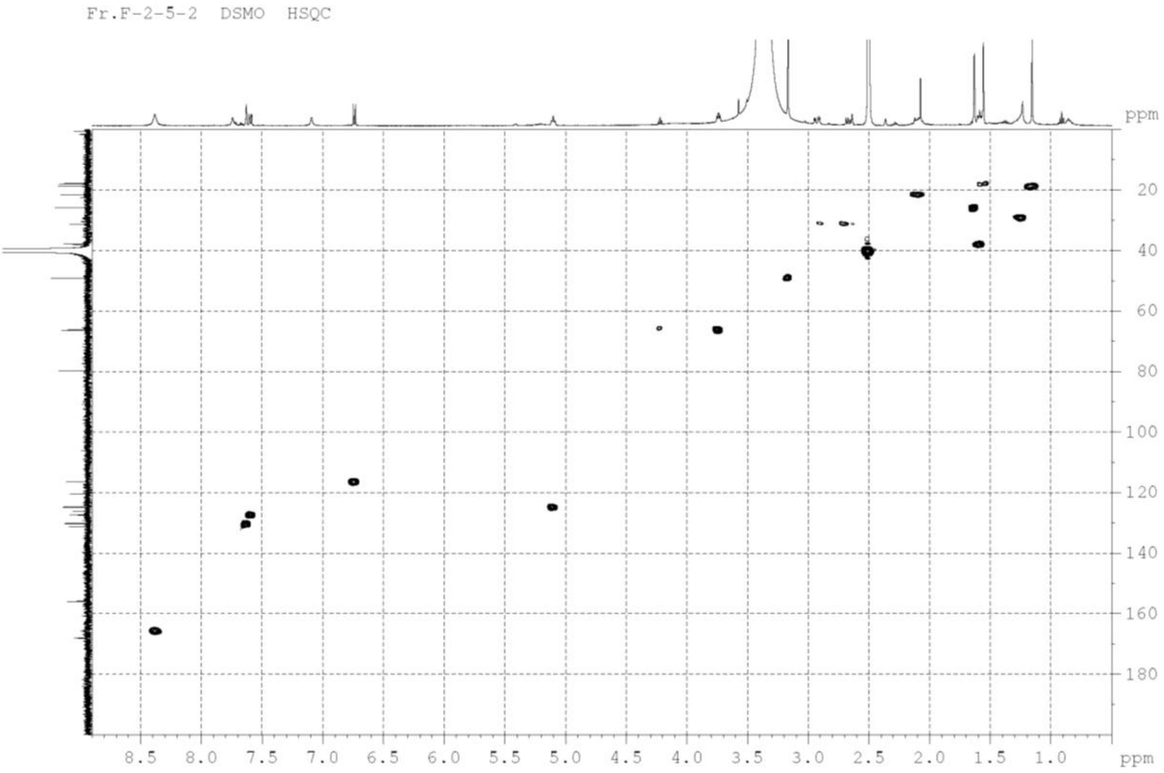

Figure S7. COSY spectrum of compound **1** in DMSO-d_6.

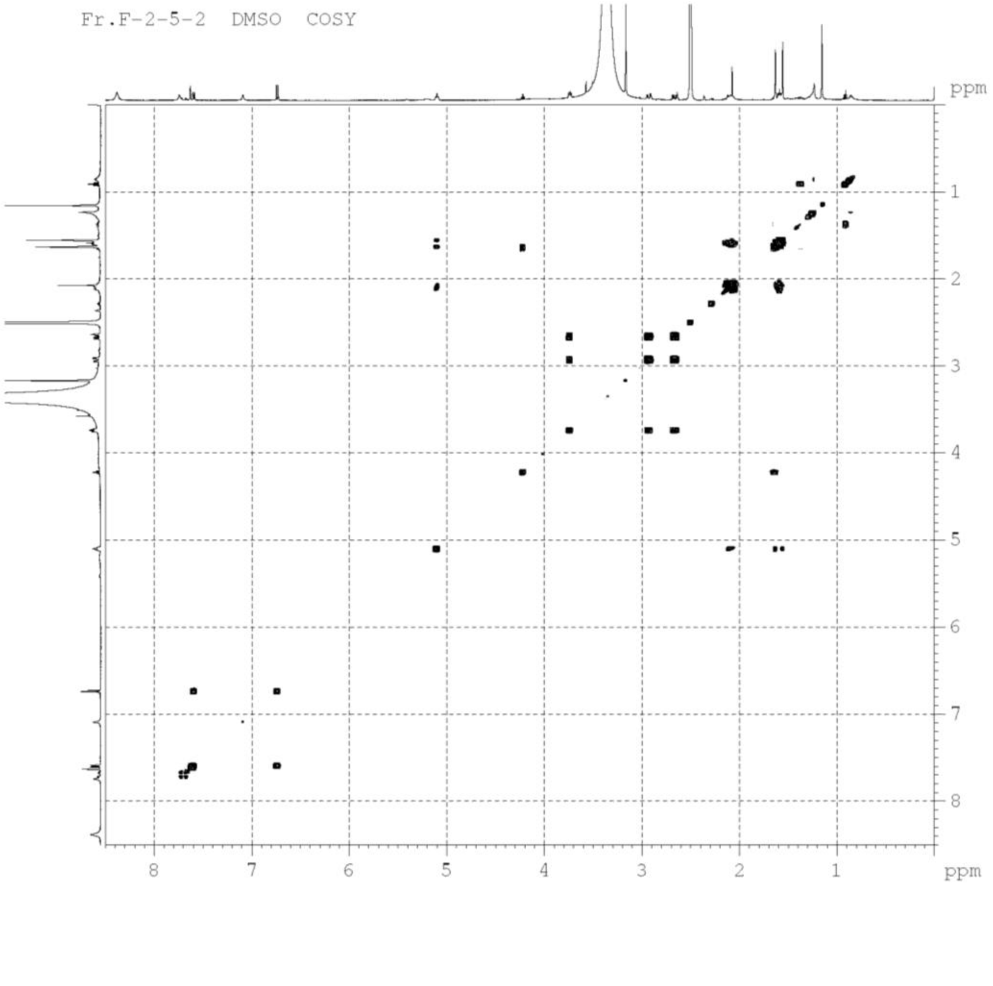

Figure S8. NOSEY spectrum of compound **1** in DMSO-d_6.

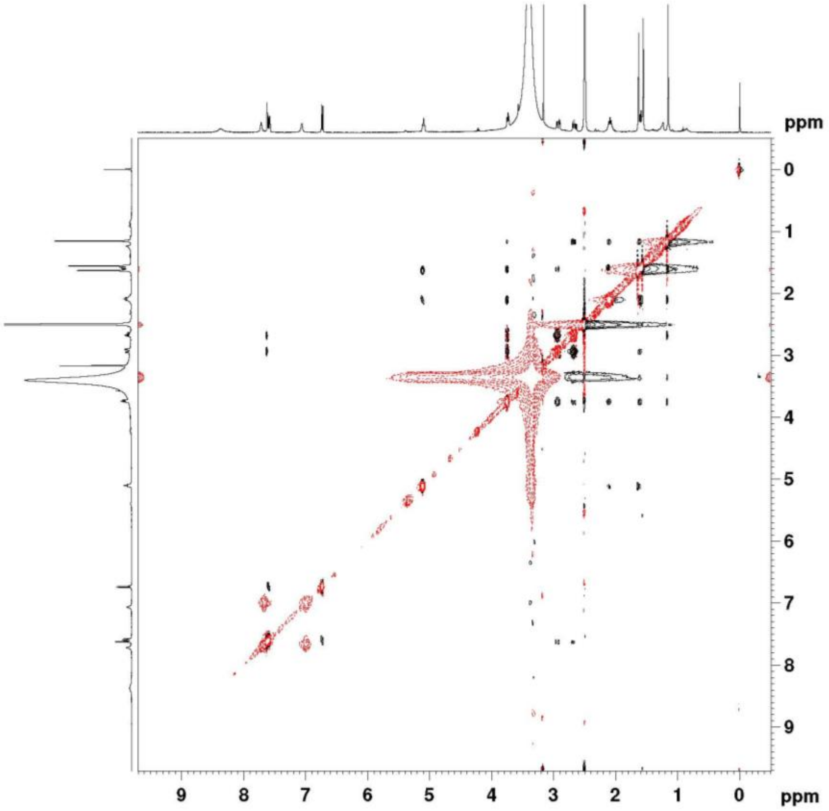

Figure S9. CD spectrum of compound **1**.

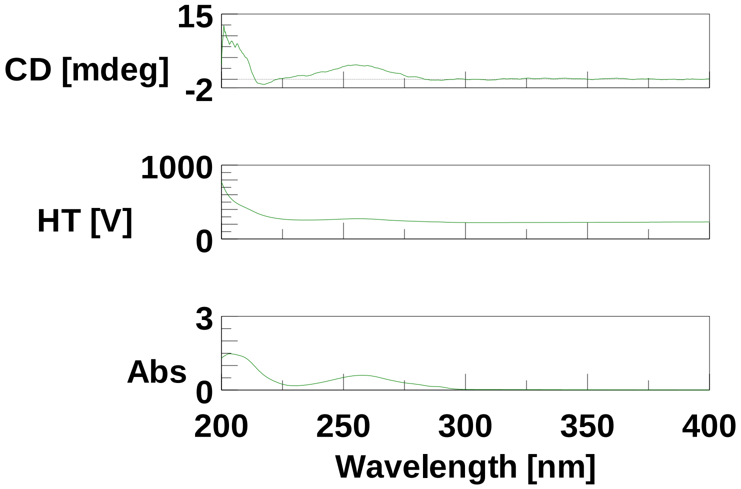

Figure S10. HRESIMS spectra of compound **2** (positve mode).

Mass	Calc. Mass	mDa	PPM	DBE	i-FIT	Norm	Conf(%)	Formula
406.2210	406.2230	-2.0	-4.9	7.5	23.6	0.280	75.55	C22 H32 N O6
	406.2171	3.9	9.6	16.5	24.7	1.435	23.82	C29 H28 N O
	406.2288	-7.8	-19.2	-1.5	28.3	5.063	0.63	C15 H36 N O11

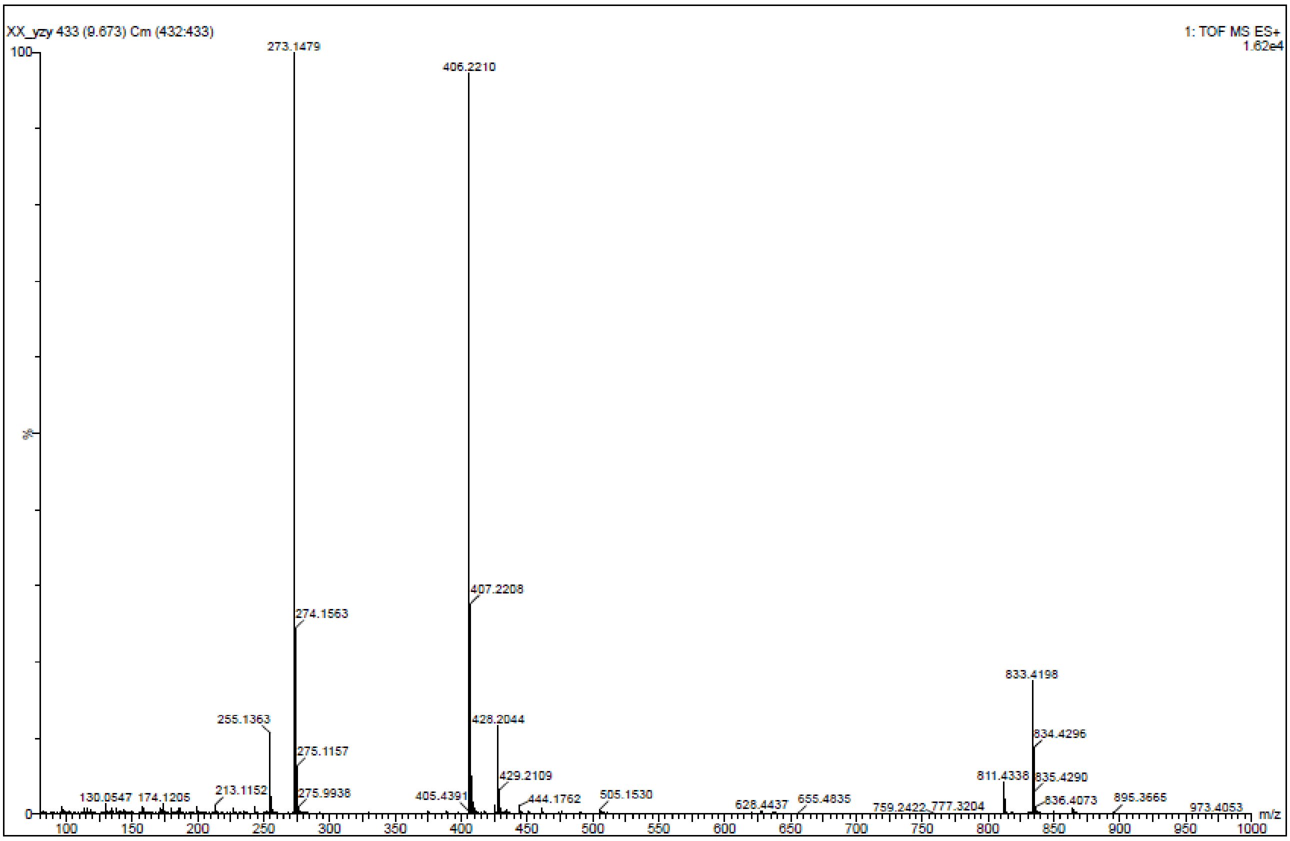

Figure S11. UV spectra of compound **2** (online detection by HPLC).

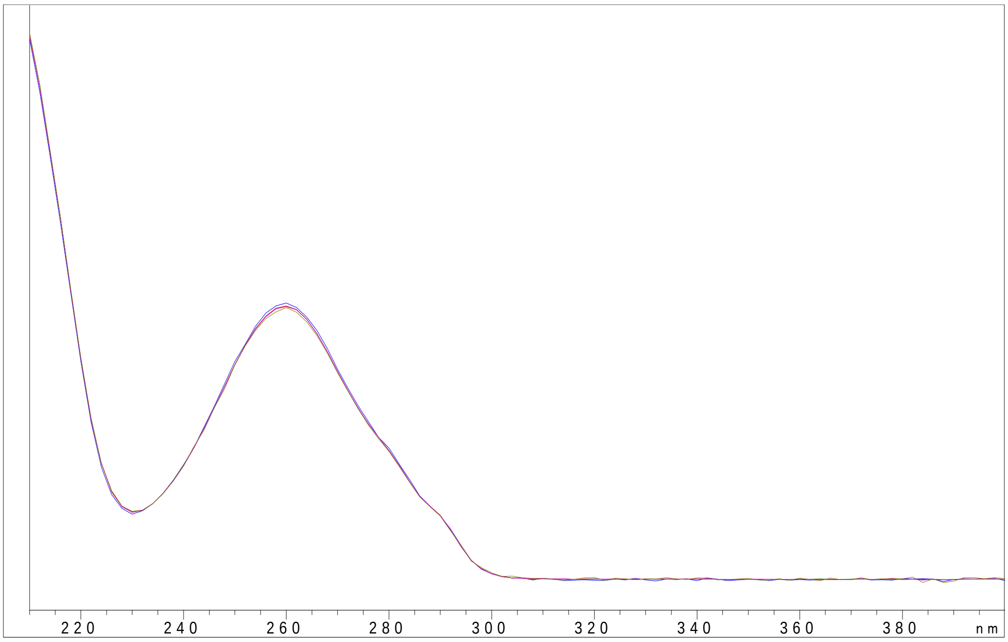

Figure S12. ^{1}H NMR spectrum of compound **2** in DMSO-d_6.

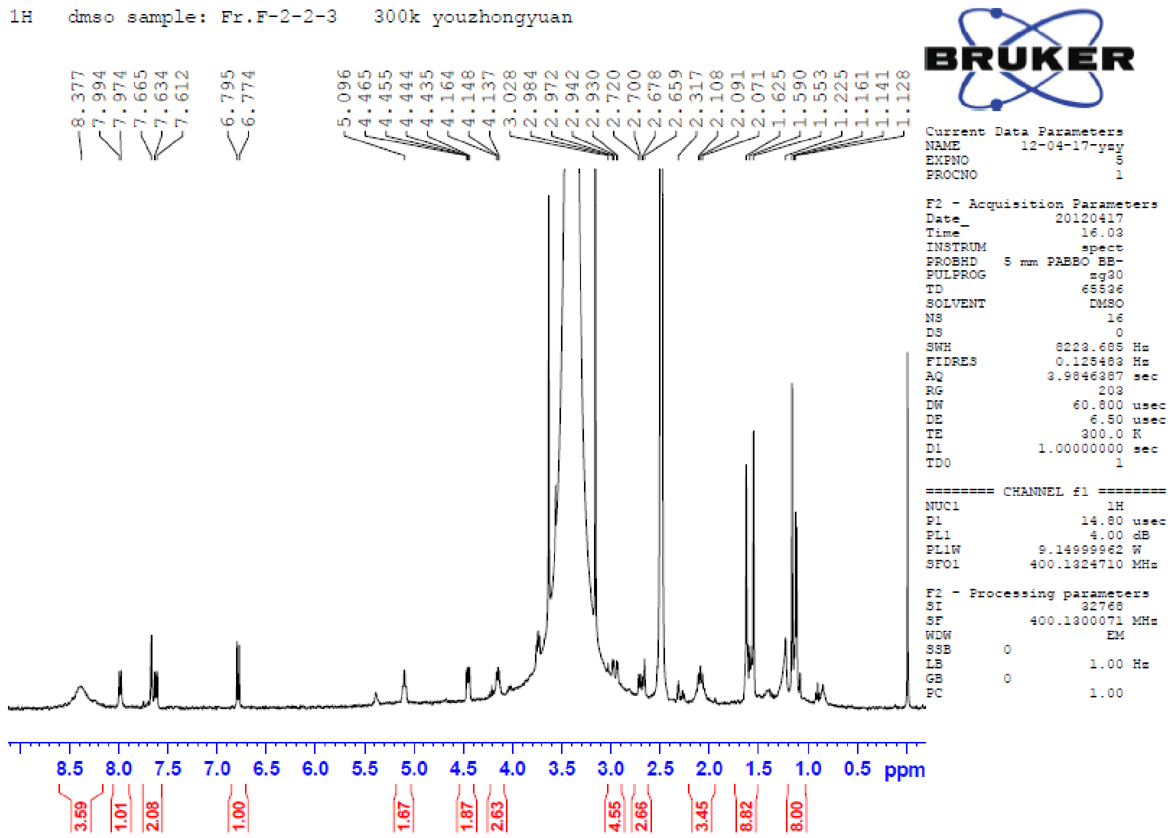

Figure S13. ^{13}C NMR spectrum of compound **2** in DMSO-d_6.

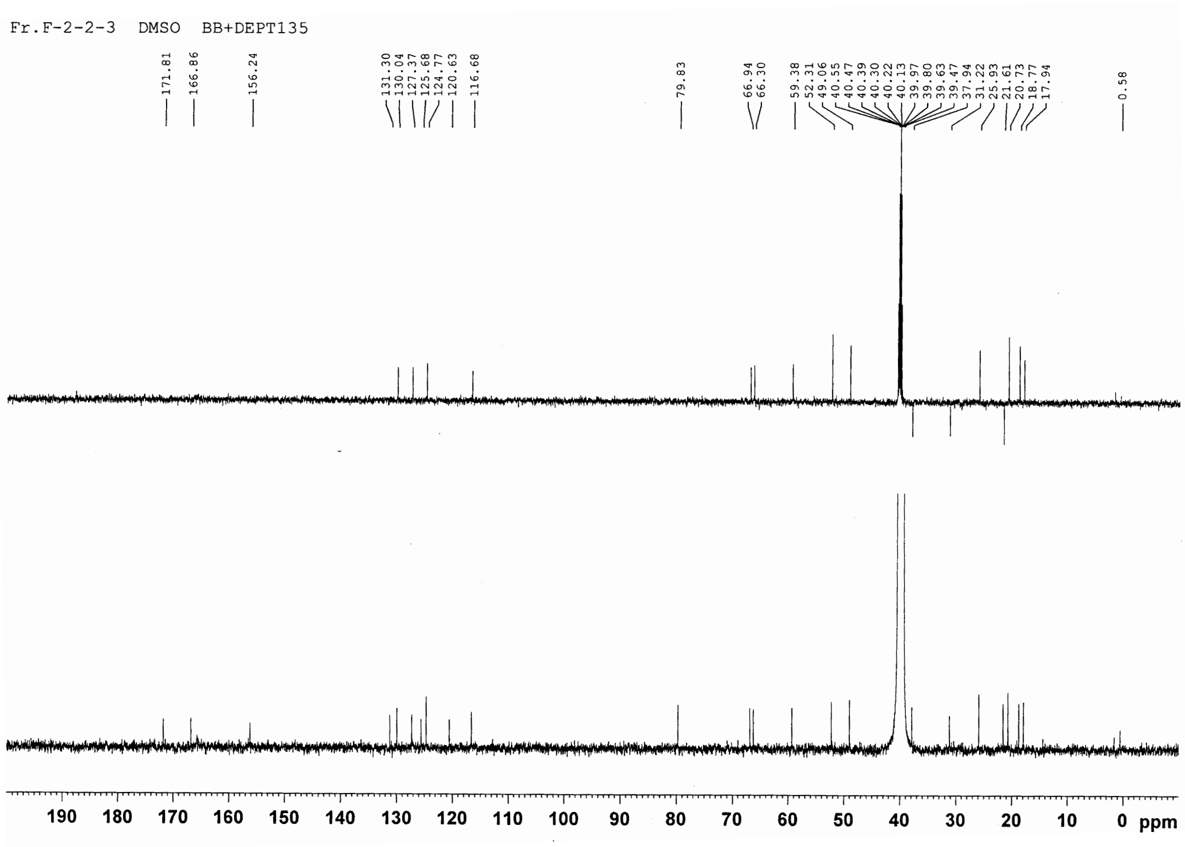

Figure S14. HMBC spectrum of compound **2** in DMSO-d_6.

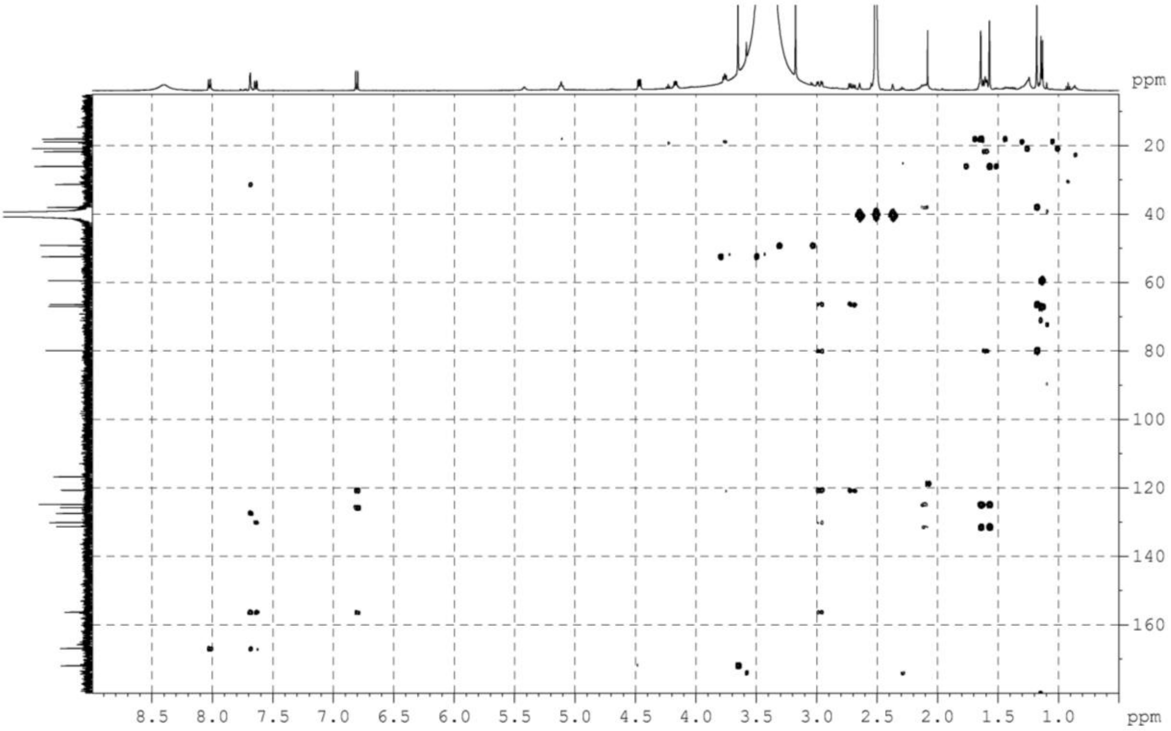

Figure S15. HMQC spectrum of compound **2** in DMSO-d_6.

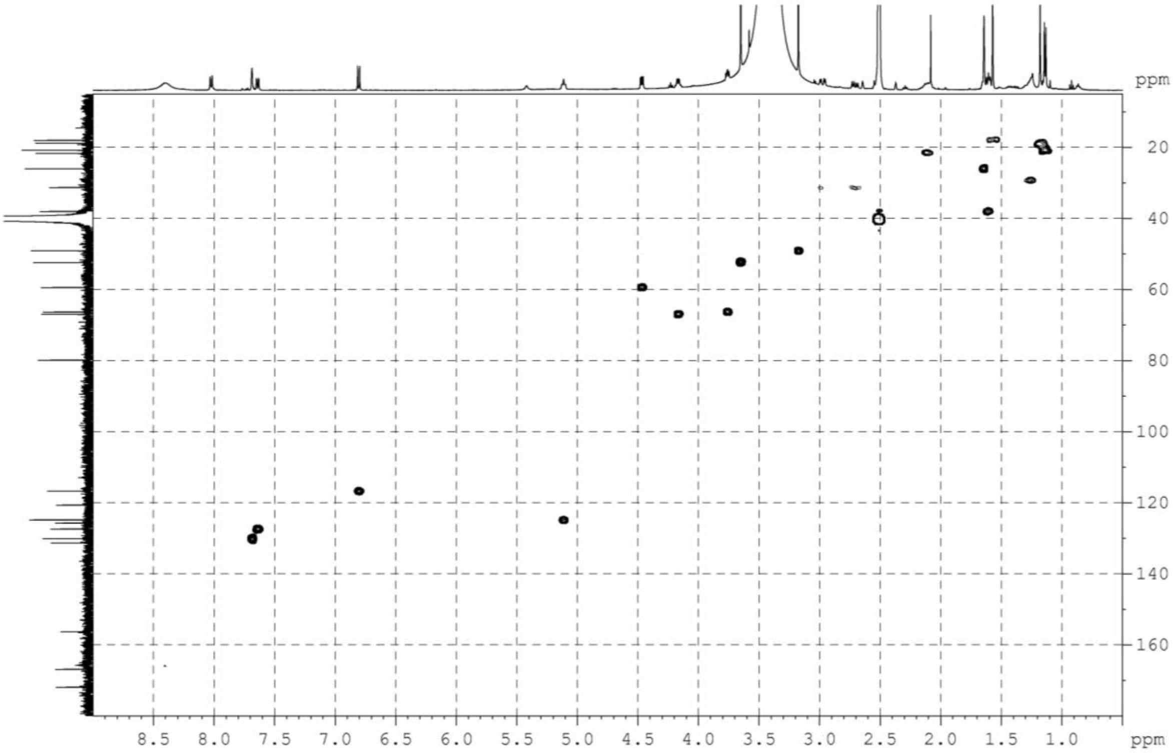

Figure S16. COSY spectrum of compound **2** in DMSO-d_6.

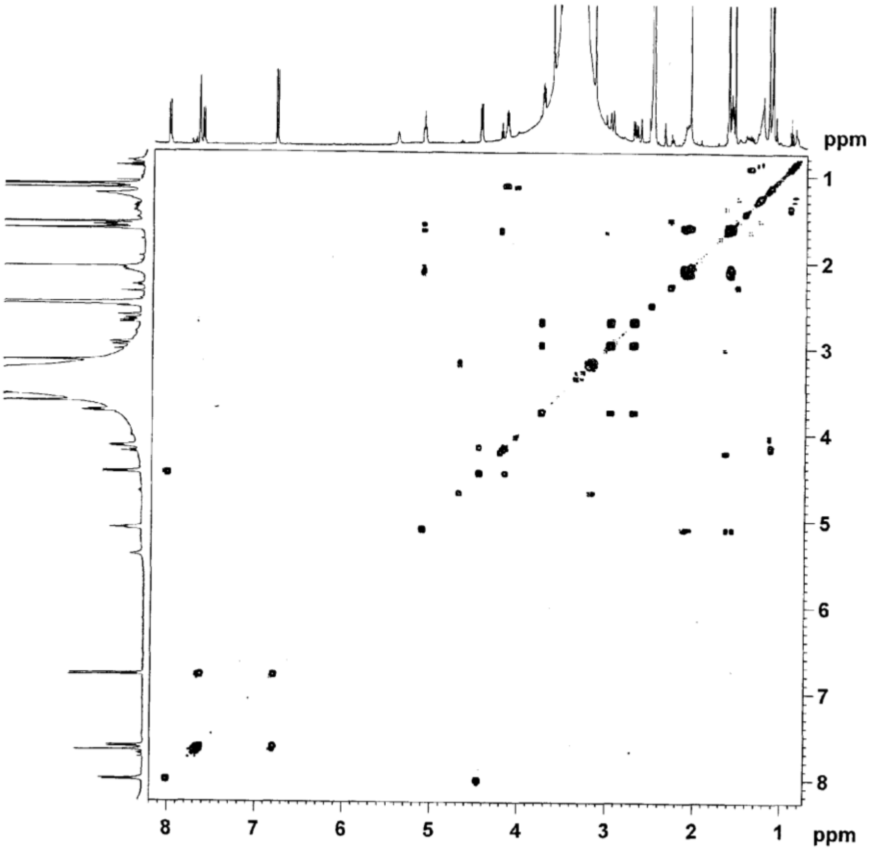

Figure S17. ROSEY spectrum of compound **2** in DMSO-d_6.

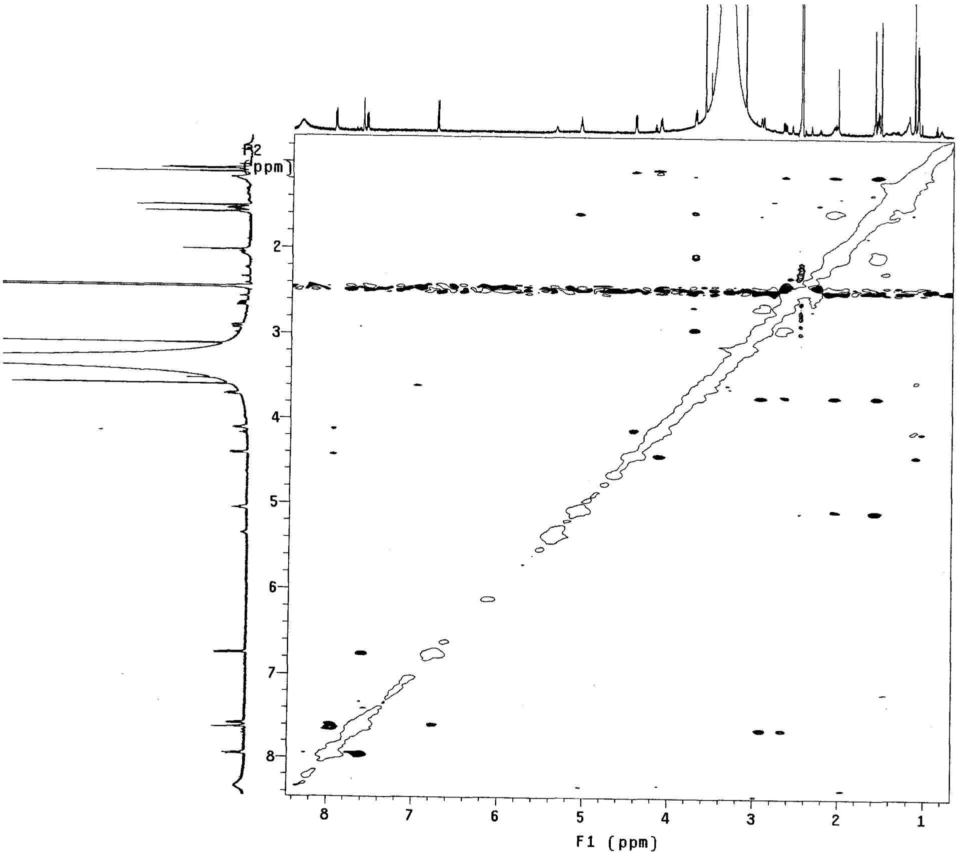

Figure S18. CD spectrum of compound **2**.

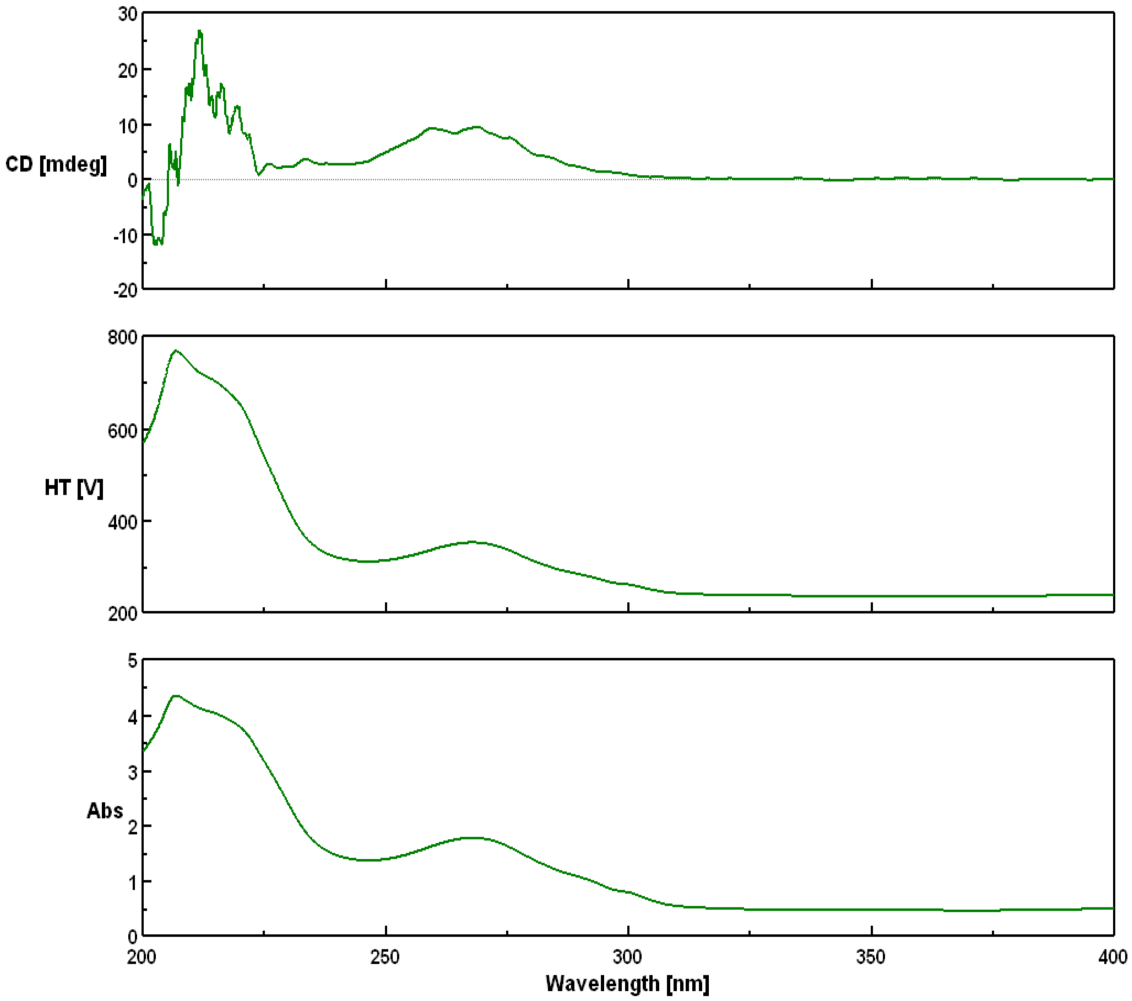

Supplement: Supplementary File 1 — Supplementary Materials (PDF, 1338 KB) [file marinedrugs-11-04035-s001.pdf]
